# Epigenetic regulation of synaptic disorder in Alzheimer’s disease

**DOI:** 10.3389/fnins.2022.888014

**Published:** 2022-08-03

**Authors:** Zhiying Chen, Moxin Wu, Qin Lai, Weixin Zhou, Xiaoqing Wen, Xiaoping Yin

**Affiliations:** ^1^Department of Neurology, Affiliated Hospital of Jiujiang University, Jiujiang, China; ^2^Jiujiang Clinical Precision Medicine Research Center, Jiujiang, China; ^3^Department of Medical Laboratory, Affiliated Hospital of Jiujiang University, Jiujiang, China

**Keywords:** Alzheimer’s disease, synaptic disorders, epigenetics, memory impairment, DNA methylation, RNA interference, histone modifications, therapeutic target

## Abstract

Synapses are critical structures involved in neurotransmission and neuroplasticity. Their activity depends on their complete structure and function, which are the basis of learning, memory, and cognitive function. Alzheimer’s disease (AD) is neuropathologically characterized by synaptic loss, synaptic disorder, and plasticity impairment. AD pathogenesis is characterized by complex interactions between genetic and environmental factors. Changes in various receptors on the postsynaptic membrane, synaptic components, and dendritic spines lead to synaptic disorder. Changes in epigenetic regulation, including DNA methylation, RNA interference, and histone modification, are closely related to AD. These can affect neuronal and synaptic functions by regulating the structure and expression of neuronal genes. Some drugs have ameliorated synaptic and neural dysfunction in AD models *via* epigenetic regulation. We reviewed the recent progress on pathological changes and epigenetic mechanisms of synaptic dysregulation in AD to provide a new perspective on this disease.

## Introduction

Alzheimer’s disease (AD) is a degenerative disease of the central nervous system characterized by progressive cognitive and behavioral impairment, which seriously affects the quality of life of the elderly (Shah et al., 2016; Chhatwal et al., 2022). AD is caused by complex interactions between genetic and environmental factors and lacks effective treatment measures. Neuropathological features of AD, including synaptic loss, synaptic disorder, and impaired plasticity, may form the pathological bases behind the development of cognitive impairment (Zhu et al., 2018; Qu et al., 2021). As the structural basis of brain function, synaptic connections continue to undergo dynamic changes. During development, learning, stress, and other processes, synapses exhibit remarkable plasticity and are easily disturbed by many factors. Mounting evidence has shown that the abnormal aggregation of amyloid-beta (Aβ), tau, and other proteins can cause synaptic disorder in fragile brain regions by disturbing and prolonging long-term potentiation (LTP). As a result, there are changes in postsynaptic membrane receptors, altering synaptic plasticity, and changing the morphology and density of dendritic spines (Jeong et al., 2021). These lead to decreased synapses and eventually cause various disorders, primarily cognitive and memory failure (Zhang et al., 2008; Bufill et al., 2013). In this review, we postulate that the mechanisms underlying the abnormal aggregation of Aβ, tau, and other proteins which abrogate synaptic function may be epigenetic in nature. Epigenetics focuses on the study of changes in gene expression without any changes in DNA sequence. A study found that the *PP2A* gene encoding the protein phosphatase 2A was in a demethylated state in the hippocampus of AD patients and mice (Zhou et al., 2008). Demethylation at L309 of PP2A leads to an increase in tau protein, while reduction of methylation at L309 reduces tau hyperphosphorylation, thus reducing intersynaptic protein function. Scientists have found that micro RNA (miRNA) imbalance in AD patients and models is related to amyloid precursor protein (APP) expression, production of Aβ, deposition of pathological tau protein, inflammatory response, and damage to the cytoskeleton, leading to synaptic disorder (Wang et al., 2019; Jash et al., 2020). In addition, several studies have revealed that histone acetylation is related to synaptic disorders and learning and memory disorders (Graff et al., 2014; Yamakawa et al., 2017). Therefore, epigenetic studies have opened a new chapter for exploring the pathogenesis and treatment of these disorders. This article reviews the epigenetic mechanisms and treatments regarding DNA methylation, histone modifications, and RNA modifications in synaptic dysregulation in AD.

## Synaptic disorders in Alzheimer’s disease brain pathology

In a healthy adult brain, the hippocampus and cerebral cortex functions are crucial for learning and memory. LTP plays an essential role in their maintenance. LTP is a persistent enhancement phenomenon that occurs in the signal transmission of two neurons, which can stimulate two neurons synchronously, which is one of several phenomena related to synaptic plasticity, which is the ability of synapses to change strength (Jeong et al., 2021). Sustained LTP is closely associated with increased synaptic calcium ion (Ca^2+^) levels and the enhancement of kinase activity, which maintains healthy neural activity and connections (Dudai and Morris, 2013). In pathological tissue sections of AD patients, it was found that Aβ oligomers can activate calcineurin in cell signaling pathways, leading to dendritic spine loss and resulting in LTP disorders. This triggers synaptic damage and impairs learning and memory function (Alberdi et al., 2010). It was also found that the lack of *PP2A* led to axon growth disorders in AD mouse models (Kuchibhotla et al., 2014). In the transgenic rTg4510 mice expressing tau protein with P301L mutation, mutant tau can cause dendritic spine loss and synapse collapse, causing severe cognitive impairment (Kuchibhotla et al., 2014).

Synaptic failure is a critical component of the neurodegenerative process of AD, especially in early AD (Spires-Jones and Hyman, 2014; Wu et al., 2021). Synaptic plasticity denotes changes in the efficacy of synaptic transmission in response to neuronal activity; it also promotes related structural plasticity responses exemplified by dendritic spine remodeling (Weng et al., 2011). In AD mouse models, highly expressed delta-secretase induced the formation of senile plaques and neurofibrillary tangles in the brain, causing neuroinflammation and neuron loss, leading to an apparent cognitive disorder. Overexpression of delta-secretase significantly reduced the formation of dendritic spines in wild-type hAPP/hMAPT double transgenic mice. These findings support that delta-secretase plays an essential role in AD development by influencing synaptic disorders (Latina et al., 2018).

**FIGURE 1:**
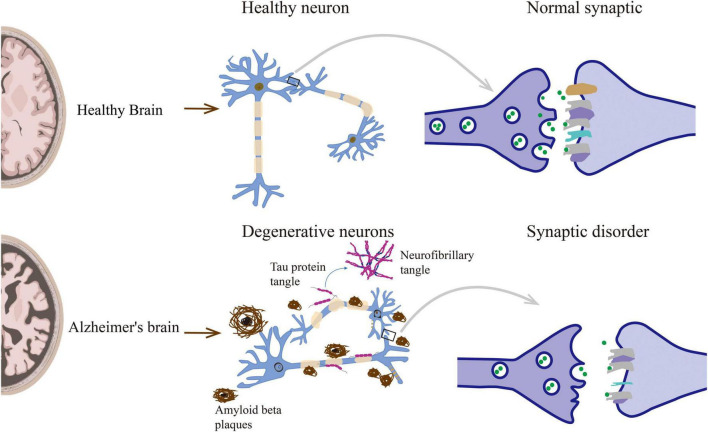
Neuropathological lesions in Alzheimer’s disease include senile plaques, neurofibrillary tangles, and other causes leading to synaptic disorders.

Synaptic disorders can directly lead to memory and behavioral dysfunction, and understanding its changes and regulatory mechanisms is essential. Therefore, there are significant synaptic disorders in AD, including synapse loss, changes in the morphology and number of dendritic spines, and synaptic transmission disorders (Figure 1).

## The complex mechanism of synaptic dysregulation in Alzheimer’s disease

There are many causes of synaptic dysregulation in AD, including cytoskeleton structure destruction caused by excessive deposition of Aβ and tau proteins, neurotransmitter system disorder (Bukke et al., 2020). Other factors include an inflammatory immune response (Uddin et al., 2021), lipid and issues of biological energy metabolism (Xiang et al., 2015). Oxidative stress, and epigenetic alterations also play a role in synaptic dysregulation (Mastroeni et al., 2015). Epigenetic regulation disorders play a crucial role in the pathogenesis of AD, including abnormal DNA methylation, histone modification, and RNA interference, and have essential value in the pathogenesis of cellular metabolic and synaptic disorders (Van den Hove et al., 2014; Nikolac et al., 2021).

Excessive Aβ binding to N-methyl-D-aspartate receptors depolarizes postsynaptic neurons, increases Ca^2+^ influx, activates multiple Ca^2+^-dependent signaling pathways, and changes downstream-related proteins, including calpain and calcineurin enzymes (De Felice et al., 2009). Inactivation of PP2A and increased Fyn levels lead to in mitochondrial permeability and functional breakdown changes, which ultimately leads to synaptic disorder (De Felice et al., 2009; Kamat et al., 2016). It is also involved in the inflammatory immune response, oxidative stress, and other causes of nerve cell metabolism disorders. It subsequently leads to synaptic disorder resulting in learning and memory disorders. However, the initiating factors that induce synaptic lesions include genetic and environmental factors. Individuals with abundant Aβ and tau protein expression have not yet developed AD, and approximately 1/2 of identical twins have AD (Gatz et al., 2006).

Furthermore, *APP*, beta-site amyloid precursor protein cleaving enzyme (*BACE*), and presenilin-1 (*PSEN1*) gene mutations are common in AD patients, and similar promoter mutations have different clinical manifestations in some cases. As a result, *APP*, *BACE*, and *PSEN1* genes contain controllable methylated CpG sites (Ko et al., 2015; Tecalco-Cruz et al., 2020). Autopsy of AD patients revealed that the *APP* gene in the cortex was completely demethylated. However, no similar findings were noted in the healthy controls or patients with Pick disease. *In vitro* studies have found that hypomethylation caused by folic acid deprivation enhances the expression of *BACE* and *PSEN1* genes, which returns to normal after folic acid supplementation (Fuso et al., 2008).

Therefore, we believe that epigenetic regulation is involved in AD neuropathological processes under the influence of the environment and other factors. Epigenetic regulation of synaptic function in AD is also involved in regulating synaptic disorders and plasticity and has an essential impact on learning and cognitive functions.

## Epigenetic regulation of synaptic dysregulation in Alzheimer’s disease

### DNA methylation

DNA methylation is one of the essential methods for appearance modification. Its action mechanism involves cytosine methylation in the cytidine phosphate dinucleoside at the CpG site under the action of DNA methyltransferase, which converts cytosine to 5-methylcytosine (5mC) in the presence of methyl donor S-adenosylmethionine (SAM). Studies have shown that DNA methylation can cause changes in chromatin structure, DNA conformation, DNA stability, and DNA-protein interactions, thereby controlling gene expression.

Chouliaras and colleagues conducted a genome-wide DNA methylation analysis in the hippocampal tissues of 10 AD patients and 10 healthy controls (Chouliaras et al., 2013). They found that the levels of 5mC and 5-hydroxymethylcytosine (5hmC) were significantly lower in AD patients than in the healthy controls, and the methylation levels of the entire genome decreased. Another study used gas chromatography-mass spectrometry combined technology to examine the entire genome of the superior temporal gyrus, middle temporal gyrus, hippocampus, parahippocampal gyrus, cerebellum, and inferior parietal lobe in brain tissues of AD patients at different stages. They found that the brain regions affected by AD showed a genome-wide decrease in 5mC and 5hmC levels during the early stage of AD (Ellison et al., 2017). The first complete epigenomic association study of AD identified 948 CpG sites for 918 unique genes associated with late-onset AD, with a mean difference in methylation of 2.9% (Lashley et al., 2015; Ellison et al., 2017). Further data analysis showed that compared with the healthy controls, the promoter region of the *TMEM59* gene was significantly hypomethylated in AD patients, resulting in high gene expression and highly specific inhibition of APP protein separation from the Golgi apparatus, resulting in synaptic disorder. However, another study showed that there was no significant difference in the level of methylation between brain regions (entorhinal cortex, auditory cortex, and hippocampus) and blood methylation between healthy elderly groups and AD patients (Furuya et al., 2012). In fact, this may also be related to the detection technology of DNA methylation. Because different detection methods and analysis methods will lead to differences in results. At present, the main methods are bisulfite sequencing, restriction endonucled-based sequencing and targeted methylation site sequencing. The results are most reliable only when the CpG island, promoter region, coding region, open chromatin and enhancer region are all covered (Meissner et al., 2005; Cokus et al., 2008; Rauch et al., 2008).

In a study by Liu et al. published in 2016, AD transgenic mice were fed with a folic acid-deficient or regular diet. They found that folic acid increased the methylation of *Psen1* and *App* promoters in AD transgenic mice (Liu et al., 2016; Tian et al., 2016). Furthermore, folic acid was supplemented in mouse hippocampal HT-22 cells. It was found that DNA methylation levels at CpG sites 2, 3, and 13 in the APP promoter increased in HT-22 cells. Additionally, when TgCRND8 mice were fed with a diet deficient in folic acid/B6/B12, SAM supplementation could inhibit DNA methylation, PSEN1 expression, and Aβ production. Therefore, folic acid supplementation can reduce the endogenous Aβ synthesis of PSEN1 and APP and may lead to increased cell viability in treated neurons after exposure to exogenous Aβ oligomers (Liu et al., 2016). Another study used 25 pairs of blood specimens from patients with AD and SH-SY5Y cells cultured in a folate-deficient medium to analyze cell viability using the 3-(4,5-dimethylthiazol-2)-2,5-diphenyltetrazolium bromide assay. The results showed that folate deficiency could increase DR4 expression due to DNA promoter hypomethylation in AD while upregulating the expression of DNA methyltransferases and inducing cell apoptosis (Wang et al., 2014).

**FIGURE 2:**
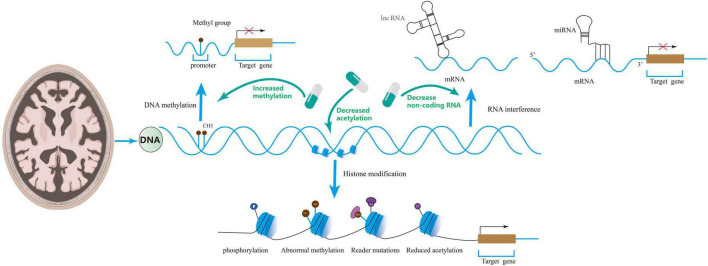
Epigenetics includes DNA methylation, RNA interference, and histone modifications that regulate synaptic disorders. Drugs can ameliorate synaptic disorders through epigenetics.

Altogether, DNA methylation modification is closely related to AD occurrence and development, and low methylation levels may cause cognitive dysfunction by impairing synaptic function. Regulation of DNA methylation can improve synaptic dysregulation in AD and cognitive function, which provides a new direction for treating these patients (see Figure 2).

### RNA interference

In recent years, RNA interference (RNAi) has been discovered as an important type of epigenetic modification. The mechanism of action is the degradation of the homologous mRNA induced by the corresponding non-coding RNA that is highly conserved in the evolutionary process, which changes gene expression without affecting the gene structure. The non-coding RNAs involved in the epigenetic regulation mechanism are mainly miRNAs. These are the most effective post-transcriptional regulatory RNA molecules that can regulate cell- and tissue-specific protein levels and play a role in the rapid, dynamic, and spatiotemporal regulation of synaptic function (Godino et al., 2015; Villa et al., 2019).

Several studies have shown that the levels of non-coding RNAs change abnormally in AD patients and animal models. Leidinger et al. (2013) sequenced the miRNAs in the blood of 48 AD patients and 22 healthy controls and analyzed the enrichment of 12 selected miRNA targets. The results showed that the levels of different miRNAs in AD patients were significantly higher than those in the healthy controls. Upregulated or downregulated miRNAs were involved in pathological changes of AD. Deng et al. (2020) found that miR-128 was upregulated in mouse models of early AD. They also found that the disorder of synaptic transmission between dentate mossy cells and somatostatin inhibitory interneurons leads to memory impairment (Deng et al., 2020). Silencing miR-128 or disrupting the binding of miR-128 and Stim2 can stimulate Stim2 expression, restore synaptic function, and reduce memory function in AD mice (Deng et al., 2020).

Zheng et al. (2021) found that miR-135a-5p was abnormally downregulated in hippocampal excitatory neurons in AD mouse models. This decrease was tau-dependent and mediated by Foxd3. It also increased Rock2 activity and hyperphosphorylation of Add1 at Ser726, leading to dendritic abnormalities and memory impairment and blocking the phosphorylation of Add1 at Ser726, overexpression of miR-135a-5p, or silencing Rock2 effectively rescued synaptic disorders and memory impairment in AD mice (Zheng et al., 2021).

Similarly, microarrays and RNA-seq studies have found significant changes in the expression of long non-coding RNAs (lncRNAs) in the AD brain compared with those in control brain samples (Magistri et al., 2015; Zhou and Xu, 2015). For instance, an increase in lncRNA-51A has been reported in AD. LncRNA-51A is known to modulate the splicing of sortilin-related receptor 1, an essential gene for traffic and recycling of the β-amyloid precursor, reducing the synthesis of sortilin-related receptor 1 variant A. In bioinformatics/reannotation studies supporting the interplay between lncRNAs and AD, it was predicted that lncRNA sequences were associated with the genes encoding APP, ApoE, PS-1, and PS-2 (Holden et al., 2013). Studies have also found that the expressions of lncRNAs n336694 and miR-106b were elevated in an AD mouse model (Huang et al., 2017).

LncRNAs and miRNAs are related to pathological tau protein deposition, Aβ production, synaptic plasticity, cytoskeletal damage, and APP expression in AD patients and animal models. These studies have revealed that RNAi can participate in AD pathological changes, affect synaptic dysregulation in AD and memory impairment, and provide a new direction for diagnosis and treating AD patients.

### Histone modifications

Histone modifications include histone acetylation, phosphorylation, methylation, ubiquitination, ADP ribosylation, and other forms (Qin et al., 2020; Adithya et al., 2022). Many studies have suggested that histone modifications have a corresponding effect on synaptic disorders and are related to neurodegenerative diseases such as AD (Rao et al., 2012).

Narayan and colleagues compared the AD group with healthy control brain tissues (Narayan et al., 2015). They found that the levels of acetyl histone H3 and acetyl histone H4 in the brain tissues of AD patients were significantly increased after death. These were found in the immunopositive cones of AD brain neurons. Graff et al. (2012) found that histone deacetylase 2 (HDAC2) expression in the hippocampus of AD patients increased. Its overexpression may be related to dendritic spine density, decreased number of synapses, synaptic plasticity, and memory (Graff et al., 2012). Another study also found that HDAC2 overexpression induced AD-like tau hyperphosphorylation and aggregation, which were accompanied by a loss of dendritic complexity and spine density (Liu et al., 2017).

Disruption of histone phosphorylation of neurotransmitter receptor promoters is considered one of the causes of impaired memory function in AD (Xuan et al., 2015; Lee et al., 2020). Anderson et al. (2015) used the 5XFAD mouse model of rapid amyloid deposition to study H3 phosphorylation at S57 and T58. The results showed that the phosphorylation of S57 and T58 on histone H3 was lower in the 5XFAD model of amyloid deposition compared with the wild-type controls. This was also considered to cause synaptic damage; however, its specific mechanism requires further investigation (Sze et al., 2001; Zhao et al., 2004; Lee et al., 2020).

Studies have found that histone methylation and ubiquitination were related to regulating gene expression in the nervous system. Through western blot analysis and immunofluorescence staining combined with confocal microscopy analysis, Lee et al. (2020) found that H3K9me3-mediated heterochromatin concentration increased in the cerebral cortex after death in sporadic AD, and the downregulated genes were related to synaptic function. Among these, methylation of H2B residue K108 and H4 residue R55 were significantly reduced by 25 and 35%, respectively. Mutations in JARID1C, a highly expressed histone demethylase in the brain of adult rats, lead to memory impairment, and the JARID1C loss-of-function lead to the shortening of dendritic filament and dendritic spines (Mackowiak et al., 2014). In addition, ubiquitination of K120 on H2B increased by 91%, and the acetylation of the region near the N-terminus of H4 was also significantly reduced (Anderson and Turko, 2015). These results show a significant positive correlation between ubiquitin load and histone modifications. HDAC promotes the deacetylation of histones, thereby promoting the tight binding of histones and DNA and negative regulation of learning and memory abilities in multiple brain regions.

Rao et al. (2012) measured gene-specific CpG methylation, global DNA methylation, and histone modification in the frontal cortex after death in AD patients (*n* = 10) and their age-matched healthy controls (*n* = 10). The results showed that the overall phosphorylation of histone H3 was increased in the patients’ brains. Increased methylation levels of histone H3 in mice with Aβ in the prefrontal cortex in AD transgenic mice have been reported (Pattaroni and Jacob, 2013). Millan and colleagues suggested that histone methylation significantly impacts synaptic plasticity (Millan et al., 2021). Interestingly, some studies have found that the use of HDAC inhibitors can promote the course of Alzheimer’s disease by changing the acetylation status of chromatin and other non-histone proteins, regulating the expression of target genes, inducing neuronal apoptosis and autophagy (Thomas and D’Mello, 2018; Gupta et al., 2020). Therefore, aside from their essential roles in gene transcription, HDAC proteins are now known to deacetylate a large and ever-growing number of non-histone proteins (Spange et al., 2009).

The results of these studies suggest that excessive histone acetylation and phosphorylation are involved in regulating the content of Aβ in the brain, which in turn is directly related to synaptic disorder. Even non-histone regulation may be involved in the course of AD.

## Modulation of epigenetic regulatory mechanisms as a therapeutic target in Alzheimer’s disease

Synaptic disorders in AD can be improved by regulating epigenetic mechanisms. In 2019, Roman and others reported that reduced levels of SAM induce DNA demethylation, leading to overexpression of pathologically related genes in AD (Roman et al., 2019). Methionine reacts with ATP to form SAM, SAH, and homocysteine. According to a recent study, moderately elevated homocysteine increased the relative risk of dementia in older adults from 1.15 to 2.5 times, with population-attributable risk rising from 4.3 to 31% (Raszewski et al., 2016). Therefore, homocysteine reduction therapy with vitamin B may significantly slow the rate of brain shrinkage and cognitive decline in older adults by upregulating DNA methylation (Smith et al., 2018). Li et al. (2016) showed that folic acid upregulated DNA methylation in N2a-APP cells and AD transgenic mice and analyzed the role of folic acid in JAK-STAT and LTD signaling pathways and its value in AD treatment. Therefore, increasing methylation levels and activities may represent a potential therapeutic strategy for ameliorating AD (Wang et al., 2013; Li et al., 2016).

MiRNA-206 is highly expressed in APP/PSEN1 transgenic mice, mainly in the plasma, cerebrospinal fluid, and hippocampus. It is associated with the downregulation of BDNF, which has also been observed in AD patients (Tian et al., 2014; Silvestro et al., 2019). Intranasal AM206, an antagonist of miRNA-206, was found to block the Aβ pathogenicity (1-42) and increase BDNF levels, synaptic density, and neurogenesis (Satoh, 2012). Similarly, Wang and others found that melatonin supplementation could reverse EPACs/miR-124/Egr1 signal conduction and restore the amplitude and slope of field excitatory postsynaptic potential (Wang et al., 2013). They also found it can improve the density of spines and percentage of mushroom spines in CA1 neurons and reduce synaptic disorder and memory deficits in an anisodamine-induced AD model (Wang et al., 2013).

Cao et al. (2020) found that the H3K4me3 histone marker and catalytic enzyme were significantly increased in the prefrontal cortex in AD patients and mice models. Subsequently, introducing a specific Sgk1 inhibitor into mice reduced excessive phosphorylated tau proteins, restored the prefrontal cortex and glutamate synaptic function, and improved the memory defects in AD mice (Cao et al., 2020). Additionally, several AD mouse models have been treated with HDAC inhibitors, such as sodium butyrate, trichostatin A, and valproic acid, showing improvements in learning and memory, some resulting from reduced Aβ levels (Sung et al., 2013). A phase 3 clinical trial of valproate-regulated histone acetylation-modifying therapy in dementia did not improve outcomes in dementia patients (Tariot et al., 2011). Insufficient evidence supports valproic acid in treating dementia for cognitive, psychiatric symptoms or disease-modifying (Liu and Wang, 2020). The failure of these clinical trials may be due to insufficient plasma concentrations of sodium valproate to engage relevant molecular targets, particularly glycogen synthase −3β. In practice, patients cannot use higher doses of valproate.

Chatterjee et al. (2018) identified interaction partners of target proteins and epigenetic targets through the human protein interaction network. These networks include DNA methyltransferase inhibitors, histone deacetylase inhibitors, histone acetyltransferase modulators, histone methyltransferase inhibitors, and histone demethylase inhibitors. These can be used as epigenetic drugs to reverse the epigenetic changes and correct memory deficits and behavioral abnormalities after improved synaptic disorder in AD, which can help develop new drugs for AD treatment (Ganai et al., 2016; Chatterjee et al., 2018). Some researchers have also launched a phase I clinical trial of Vorinostat (an HDAC inhibitor) in the treatment of mild Alzheimer’s disease. But so far there is no clear conclusion of safety and efficacy. Therefore, we look forward to further large-scale clinical studies.

## Prospects

In summary, epigenetic modifications play an essential role in the occurrence and development of synaptic dysregulation in AD. Modification abnormalities, such as DNA methylation, histone modification, and RNA interference, lead to abnormal transcription of AD-related genes, resulting in dysfunction of related proteins and enzymes, synaptic disorder, and memory behavior disorders. In theory, diseases caused by epigenetic modifications can be reversed with drugs. For example, folic acid supplementation can increase DNA methylation and inhibit Aβ deposition. Melatonin can reverse EPACs/miR-124/Egr1 signaling, improving the density of spines and the percentage of mushroom spines in CA1 neurons. Sgk1 inhibitors can reduce hyperphosphorylated tau proteins by regulating H3K4me3 and restoring PFC glutamate synaptic function, providing a new approach for AD treatment. However, the clinical application of epigenetic drugs may be a long way off due to the unknown side effects and complex co-regulatory mechanisms that limit their development. Therefore, further research on the epigenetic mechanisms in the pathogenesis and development of synaptic dysregulation in AD is essential to discover safe and specific drugs that regulate epigenetic mechanisms.

## Author contributions

ZC and MW: conception and design and administrative support. ZC, MW, QL, WZ, XW, and XY: provision of study materials. ZC, MW, and QL: collection and assembly of data. ZC, XW, and XY: data analysis and interpretation. XY: revision of the final version. All authors: manuscript writing and final approval of the manuscript.

## Abbreviations

Aβ: amyloid-beta
BACE: beta-site amyloid precursor protein cleaving enzyme
AD: Alzheimer’s disease
APP: amyloid protein precursor
HDAC2: histone deacetylase 2
lncRNAs: long non-coding RNAs
LTP: long-term potentiation
miRNA: micro RNA
PSEN1: presenilin-1
RNAi: RNA interference
SAM: S-adenosylmethionine
Ca^2+^: calcium ions.
